# Noise-Resilient Whitened Domain Adaptation for Intelligent Mechanical Fault Diagnosis Under Non-Stationary Sensor Signals

**DOI:** 10.3390/s26103222

**Published:** 2026-05-19

**Authors:** Qinyue Chen, Yunxin Xie

**Affiliations:** 1 School of Overseas Education, Changzhou University, Changzhou 213164, China; 2300160401@smail.cczu.edu.cn; 2 School of Safety Science and Engineering, Changzhou University, Changzhou 213164, China

**Keywords:** intelligent mechanical fault diagnosis, degraded sensor data, unsupervised domain adaptation, non-stationary vibration signals, noise resilience, rotating machinery

## Abstract

Intelligent mechanical fault diagnosis plays a key role in maintaining rotating machinery. Although data-driven unsupervised domain adaptation methods have achieved considerable progress, their industrial applications are often restricted by low-quality sensor data. Non-stationary vibration signals and background noise easily corrupt target pseudo-labels, while conventional methods focusing on global statistical matching usually neglect local structures, leading to confirmation bias under dynamic loads. To improve diagnostic reliability, we propose a Noise-Resilient Whitened Domain Adaptation (NRWDA) framework. To handle covariance fluctuations caused by changing working conditions, a Lipschitz-bounded Temporal Whitening (LTW) module is designed as a low-pass filter. An Entropy-guided Prototype Truncation (EPT) mechanism is adopted to discard ambiguous labels and better calibrate semantic centers. In addition, a Dispersion-Adaptive Contrastive Sharpening (DACS) strategy is introduced to dynamically adjust the contrastive temperature based on predictive dispersion, thus tightening decision boundaries. The proposed method is evaluated on CWRU, PU, and MFPT datasets. The PU dataset, featuring fluctuating loads and non-stationary signals, poses a strict test, yet our model maintains its stability even at a 0 dB SNR—a condition where standard approaches usually break down. During the P0→P3 transfer task involving substantial radial force variations, NRWDA secures a 72.36% accuracy and surpasses established baselines. These findings confirm that our technique successfully isolates dependable diagnostic features from corrupted sensor measurements within actual industrial settings.

## 1. Introduction

Ensuring the reliability of industrial equipment relies heavily on the fault diagnosis of rotating machinery. Equipment failures in smart manufacturing create direct financial costs and stop production unexpectedly. Practical industrial systems still use traditional fault diagnosis methods that require people to define features from intelligent sensor signals. These features use statistical indicators described in [1] and time-frequency analysis methods proposed in [2]. The same features are processed by standard machine learning models including support vector machines from [3] and random forests introduced in [4]. More available industrial data have led to the wide use of deep learning in fault diagnosis tasks according to [5]. The overall development from manual features to deep learning is recorded in published review papers represented by [6]. Convolutional neural networks serve as the basic structure for most modern diagnostic networks. Real industrial environments involve non-stationary working conditions and constant distribution shifts. Nearly all existing diagnostic methods lose performance under these challenging conditions. They possess an exceptional capacity to autonomously extract highly nonlinear, discriminative representations directly from raw signals [7,8]. However, the empirical success of deep-learning models is directly related to this prerequisite. Such models assume that the training and deployment data are independent and identically distributed. Overcoming this sensitivity to distribution shifts is essential for reliable fault diagnosis, particularly when analyzing complex non-stationary signals that characterize modern rotating machinery.

Real-world industrial environments inevitably deviate from this assumption. Unstable operating conditions, such as dynamic loads and variable rotational speeds, along with fluctuating background noise, degrade sensor data quality and introduce substantial domain shifts. Models trained exclusively on static source domains are highly brittle under these conditions, and their performance degrades significantly when deployed in unseen and unlabeled environments, leading to unexpected system shutdowns and increased maintenance overhead. Unsupervised Domain Adaptation (UDA) can be used to tackle the problem of performance degradation and cross-condition domain shifts, and it is currently an extensively studied field for intelligent fault diagnosis [9,10].

Existing domain adaptation approaches typically fall into two broad categories. One line of work focuses on global statistical matching, using explicit alignment criteria [11] to reduce discrepancies between marginal distributions. Correlation alignment, for example, seeks to match second-order covariance statistics between domains [12]. The other category centers on local semantic alignment through self-training or prototype learning [13,14]. Such methods aim to discover implicit cluster patterns in the unlabeled target domain, supporting more precise category-wise matching. Building on these established frameworks, recent fault diagnosis models have incorporated both prototypical contrastive learning [15] and stochastic adversarial prototype alignment [16]. Although these strategies reduce dependence on target-domain labels under mild distribution shifts, they remain limited by their focus on relatively stable or slowly varying target data. When applied to non-stationary industrial scenarios, they struggle to preserve useful feature representations, as they do not account for the dynamically evolving optimization landscape.

Traditional fault diagnosis models contain structural weaknesses that hurt performance under non-stationary conditions. A key issue comes from common normalization schemes such as batch normalization [17]. Such layers are highly sensitive to unstable batch statistics caused by changing working conditions, leading to strong transient covariate shift, as noted in AdaBN [18]. This instability distorts the underlying feature metric space, preventing local alignment strategies from adapting to drifting distributions. We term this problem the global–local disconnect. In real industrial scenarios, frequent speed fluctuations further worsen this instability. Diagnostic systems then cannot track shifting features, leading to large prediction errors that reduce system resilience required for robust operation.

Under unsupervised domain adaptation, prototype-guided alignment is especially prone to pseudo-label noise during early training stages. As prototypes are updated iteratively, incorrect predictions are incorrectly treated as trustworthy signals. This creates a harmful feedback loop of confirmation bias, a well-documented issue in self-training frameworks [19]. As a result, the degraded diagnosis model often assigns overly confident normal labels to unseen fault types, which poses significant risks to equipment safety.

A further drawback of current alignment loss functions is their dependence on fixed regularization terms. Such static penalties are not sufficiently effective for distinguishing difficult negative samples during training. Such negligence results in boundary overlap between fine-grained, easily confused fault types, a long-standing difficulty in decision-boundary alignment [20]. As a consequence, the diagnostic model is unable to differentiate between essential early-stage faults and harmless noises. Frequent false alarms are triggered, preventing operators from identifying the optimal maintenance time, and this reduces the overall reliability of the condition monitoring system.

Beyond algorithmic accuracy, industrial reliability depends heavily on managing sensor instrumental errors and the asymmetric consequences of misclassification. A missed equipment failure typically induces far more catastrophic losses than a routine false alarm. With diagnostic models increasingly acting as autonomous agents, their safe deployment requires intrinsic alignment with industrial standards rather than mere reliance on external constraints [21]. To address these operational realities, Tukeev et al. [22] designed a multi-stage condition-based maintenance logic that moves past static thresholding. By assessing unconditional control-reliability indicators, their approach coordinates operability and decision-making regions through repeated-measurement averaging, confirmation logic, and multi-fidelity channel fusion. Although our proposed framework concentrates on representation alignment, it aligns directly with this system-level objective: securing a dependable decision boundary against imperfect measurements and target domain interference.

To overcome the difficulties of metric instability, prototype contamination and boundary adhesion, an effective unsupervised domain-adaptation framework named Noise-Resilient Whitened Domain Adaptation (NRWDA) is proposed in this paper. Deviating from the traditional consecutive alignment framework, this study introduces a highly integrated tight-coupled joint global debiasing and local boundary sharpening model. Under this coordination mechanism, a Lipschitz-bounded Temporal Whitening module first establishes a statistical stability base. On this basis, Entropy-guided Prototype Truncation can achieve macroscopic semantic alignment. Consequently, Dispersion-Adaptive Contrastive Sharpening fine-tunes the microscopic decision boundaries. Combining various methods to obtain and group the domain-invariant feature set, we address complex non-stationary signals and drift, so as to ensure the robust and reliable operation of rotating machinery.

The main contributions of this paper are summarized as follows:

- A stable unsupervised domain-adaptation method called NRWDA is proposed to diagnose faults in rotating machinery. Transient metric instability, pseudo-label confirmation bias and fine-grained boundary adaptation can be resolved by one system at the same time.
- Lipschitz-bounded Temporal Whitening (LTW) is then added to address the global–local dissociation problem explicitly. Under the imposition of a Lipschitz constraint that limits the speed of statistical divergence, it functions as a temporal low-pass filter for suppressing short-term covariance mismatch fluctuations. This provides a stationary metric space for subsequent local alignment.
- We develop an Entropy-guided Prototype Truncation (EPT) mechanism that can effectively address confirmation bias. By combining a time-decaying source-anchored warm-up scheme, the proposed model realizes the continuous safe loss decay of ambiguous pseudo-labels, thereby ensuring reliable macroscopic semantic alignment under intense target-domain noise constraints.
- We propose the design of a Dispersion-Adaptive Contrastive Sharpening (DACS) method to solve fine-grained feature attachment problems. Deviating from the traditional static contrastive objective, an adaptive temperature function with dynamic direction can implicitly whiten gradients automatically. It successfully separates strongly confused fault categories to eliminate early-stage false alarms.
- Several non-stationary fault-diagnosis benchmark systems are used to test the performance of the presented NRWDA approach. Empirical results show that the above structures are better in the case of drastic changes caused by operation and obvious background interference.

## 2. Related Work

### 2.1. Deep Learning and Normalization Strategies in Fault Diagnosis

Convolutional and residual networks are widely employed to learn end-to-end feature extraction from raw vibration sensor data. According to research, these deep neural networks can improve diagnostic reliability and accuracy when faced with noisy conditions [23]. Similar advanced data-driven architectures have also demonstrated exceptional capability in broader non-destructive testing, such as extracting material properties from complex ultrasonic waves [24]. However, such achievements are usually accompanied by certain unstable underlying factors. To address this issue, several domain-oriented normalization techniques have been developed. Group Normalization [25] and Transferable Normalization [26] are representative approaches designed to adjust intermediate feature statistics across domains.

However, practical industrial applications are usually non-stationary, and these normalization methods fail to meet such needs. Most of the existing approaches focus on space-batch-level operations without taking into account how distributions change with time. High-frequency fluctuations caused by sudden operation can accumulate in the feature statistics. Because there is no temporal smoothing mechanism or Lipschitz constraint, such large-scale fluctuations spread to the lower layers. Given that updating the batch statistics without controlling for divergence can result in an unstable latent metric space, this issue severely degrades the generalization ability of the model. As soon as the underlying feature space starts to deviate uncontrollably, local alignment procedures may fail to maintain correspondence changes in representation. Due to ongoing misalignment, the distinct patterns of localization and globalization are disrupted before affecting subsequent domain recognition, and this eventually reduces the diagnostic stability required for reliable condition monitoring, as repeated bias in fault detection severely compromises the generalization ability of the diagnosis system.

### 2.2. Prototypical Domain Adaptation and Pseudo-Labeling Challenges

Unsupervised domain adaptation methods aim to reduce discrepancies across domains. Early condition monitoring approaches often used macro-level schemes such as multi-layer Maximum Mean Discrepancy [27]. However, these approaches still fail to effectively address the problem of noise under industrial environmental conditions and struggle to distinguish minor faults. More recent work has explored prototypical adaptation and entropy-oriented frameworks [14] to maintain category-level discriminability.

These methods cluster target representations close to the evolving prototypes of specific classes. Hence, their evaluation depends on the quality of the pseudo-annotated labels for these targets. To mitigate confirmation bias, curriculum learning strategies [28] and uncertainty estimation techniques like MC Dropout [29] are commonly used to refine pseudo-labels. However, in a changing environment of dynamic industries, normalization instability may perturb the early feature space. Therefore, the first prediction results may be more affected by noise. In general, the standard pseudo-labeling procedure relies on either an argmax selection or a fixed confidence threshold, which consequently leads to the omission of uncertain regions adjacent to boundary classes.

According to the entropy minimization theory [30], such rigid judgments usually classify borderline cases incorrectly as belonging to other prototypes. Subsequent modifications serve to reinforce the prior erroneous behavior, which in turn accelerates error accumulation and exacerbates confirmation bias. To solve this problem, we need to eliminate ambiguous target samples by calculating their prediction entropy and then updating the prototype.

### 2.3. Robust Learning Strategies and Contrastive Boundary Challenges

Self-supervised and contrastive learning methods have also been used to isolate noise from weak fault signals. Representative approaches, including SimCLR [31], MoCo [32], and the Contrastive Adaptation Network [33], have proven effective in boosting feature discriminability. Prototypical contrastive methods are also employed in diagnostic tasks to enlarge inter-class margins [15]. Although effective, standard contrastive losses are typically rigid. Extensions such as soft-label contrastive learning [34] attempt to provide more flexibility in dense or ambiguous latent regions.

Contrastive objectives face multiple optimization problems when applied to cross-condition fault detection. The soft label adjusts the margin, but it cannot directly eliminate the gradient corruption caused by non-stationary noise. Gradients are more likely to be affected by erroneous pseudo-labeled results obtained during previous stages of training. The distorted gradient causes the model to misclassify samples within the same fault type and pulls samples from different classes closer together. No mechanisms exist to clear or stabilize such gradients; consequently, they further narrow the margin among fault characteristics. Difficult-to-distinguish early-stage failures may be distributed across this area more easily, thereby causing more misclassification in fault diagnosis and limiting the robustness of the model in practical deployments.

Most existing domain adaptation methods focus on individual components of cross-domain fault diagnosis. Recent approaches have introduced context-enhanced graph learning [35] and statistically aligned feature augmentation [36] to reduce discrepancies between source and target distributions. While effective under fixed operating conditions, such techniques rely on stable neighborhood structures and consistent batch statistics. Under dynamic loads and non-stationary sensor signals, correlation patterns shift rapidly, and early target labels carry large uncertainty. These instabilities quickly erode alignment quality, as noisy estimates accumulate and distort the feature space before reliable semantic anchors can form.

This chain of failure motivates the NRWDA framework as a unified stabilization strategy. By restraining short-term covariance variations, LTW maintains a consistent global metric space even under fluctuating operating conditions, preventing transient signal changes from breaking downstream alignment. EPT then intervenes during prototype updates, retaining only confident target samples to avoid corruption from early ambiguous assignments. Completing the pipeline, DACS adjusts gradient updates based on local sample dispersion, separating overlapping decision regions that remain difficult to distinguish using standard alignment objectives. Together, these components suppress error propagation at each critical stage of adaptation, enabling robust diagnosis under strong noise and large domain shift.

## 3. Proposed Framework

### 3.1. Problem Formulation and Framework Overview

In cross-condition fault diagnosis, Unsupervised Domain Adaptation (UDA) transfers knowledge from a labeled source domain $D_{S}={\left\{\left(x_{i}^{s},y_{i}^{s}\right)\right\}}_{i=1}^{n_{s}}$ to an unlabeled target domain $D_{T}={\left\{x_{j}^{t}\right\}}_{j=1}^{n_{t}}$. Both domains share a label space Y={1,2,…,K}, with *K* denoting the total number of fault categories. Dynamic loads and fluctuations in speed will cause considerable distributional deviations of this range. Therefore, an efficient diagnosis system must have sufficient source-domain discriminatory power and topological robustness in the target domain.

**Figure 1:**
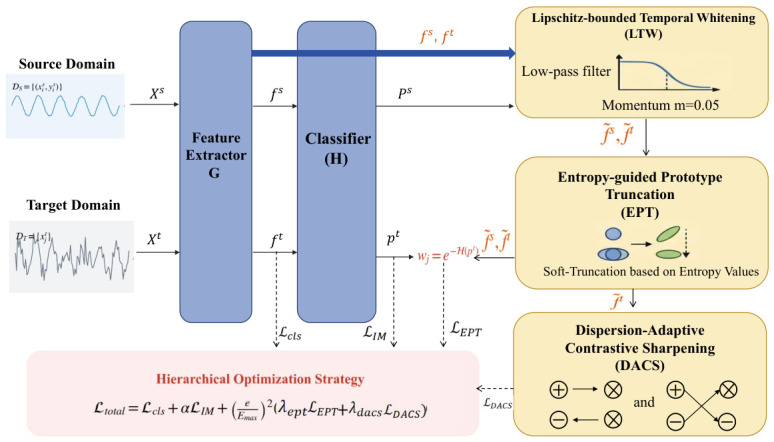
The overall architecture of the proposed NRWDA framework.

The proposed Noise-Resilient Whitened Domain Adaptation (NRWDA) system is designed to address non-stationarity in distributions and noise-polluted pseudo-labeled data. Figure 1 illustrates this architecture. A feature extractor *G* maps each input sensor signal segment *x* to a latent representation $f=G\left(x\right)\in R^{d}$. A classifier *H* then transforms this representation into a logit vector $l=H\left(f\right)\in R^{K}$.

During training with a batch size of *B*, the network extracts raw source and target features ($F_{s},F_{t}\in R^{B\times d}$) and computes their respective logits ($L_{s},L_{t}\in R^{B\times K}$). The model applies the softmax function, defined in (1), to obtain the predicted probability p(y=c|x) for the *c*th class:

(1) $$p\left(y=c|x\right)=\mathrm{Softmax}{\left(l\right)}_{c}=\frac{\exp(l_{c})}{\sum_{k=1}^{K}\exp\left(l_{k}\right)}.$$

The standard cross-entropy loss $L_{cls}$ provides explicit source-domain supervision. Based solely on this indicator, the model will suffer from confirmation bias in the target domain due to false labels in the initial stage. NRWDA solves the above problems through three collaborating modules forming a forward connection. The Lipschitz-bounded Temporal Whitening (LTW) reduces the transient variation through a low-pass filter. Entropy-guided Prototype Truncation (EPT) has class prototypes in the stabilized metric space. Dynamic Dispersion-Adaptive Contrastive Sharpening (DACS) refines the boundaries of instances dynamically.

### 3.2. LTW: Lipschitz-Bounded Temporal Whitening

Raw deep features $f\in R^{B\times d}$ often exhibit large batch-wise statistical oscillations during early cross-domain adaptation. Standard batch normalization typically employs a momentum of 0.1. This parameter choice forces features to over-adapt to current batch statistics, which exacerbates internal covariate shifts.

The LTW module reformulates feature scaling as a temporal smoothing regularization. It maintains a global running mean $\mu_{run}$ and a running variance $\sigma_{run}^{2}$, iteratively updating them via (2):

(2) $$\begin{array}{cc} \mu_{run}^{\left(t\right)} & =\left(1-m\right)\mu_{run}^{\left(t-1\right)}+m\cdot \mu_{B},\\ {\left(\sigma_{run}^{2}\right)}^{\left(t\right)} & =\left(1-m\right){\left(\sigma_{run}^{2}\right)}^{\left(t-1\right)}+m\cdot \left(\sigma_{B}^{2}+\epsilon \right), \end{array}$$

where $\mu_{B}$ and $\sigma_{B}^{2}$ represent the current batch statistics, and $\epsilon =10^{-4}$ prevents singularity. LTW projects the smoothed features (denoted uniformly as $\tilde{f}$ for both domains) via (3) using learnable affine parameters $\gamma ,\beta \in R^{d}$:

(3) $$\tilde{f}=\frac{f-\mu_{run}^{\left(t\right)}}{\sqrt{{\left(\sigma_{run}^{2}\right)}^{\left(t\right)}}}⊙\gamma +\beta .$$

LTW stabilizes the metric space and prevents statistical divergence through Lipschitz continuity. Assuming the post-activation intermediate features are bounded within a finite hypersphere, the condition $∥\mu_{B}∥\leq C$ holds for a constant C>0 across all mini-batches. Based on the iterative formulation in (2), the single-step statistical drift of the running mean can be isolated:

$$\mu_{run}^{\left(t\right)}-\mu_{run}^{\left(t-1\right)}=\left(1-m\right)\mu_{run}^{\left(t-1\right)}+m\mu_{B}^{\left(t\right)}-\mu_{run}^{\left(t-1\right)}=m\left(\mu_{B}^{\left(t\right)}-\mu_{run}^{\left(t-1\right)}\right).$$

Applying the triangle inequality to the Euclidean norm yields:

$$∥\mu_{run}^{\left(t\right)}-\mu_{run}^{\left(t-1\right)}∥=m∥\mu_{B}^{\left(t\right)}-\mu_{run}^{\left(t-1\right)}∥\leq m\left(∥\mu_{B}^{\left(t\right)}∥+∥\mu_{run}^{\left(t-1\right)}∥\right).$$

Given the topological boundary assumption $∥\mu_{B}∥\leq C$, the drift satisfies a strict Lipschitz constraint controlled by the momentum parameter *m*:

(4) $$∥\mu_{run}^{\left(t\right)}-\mu_{run}^{\left(t-1\right)}∥\leq 2cm.$$

To prevent drastic feature variations, this constraint ensures that even if a target mini-batch is contaminated by noise, the resulting perturbation injected into the global metric space cannot exceed 2Cm.

Setting m=0.05 establishes a memory window of approximately 20 mini-batches ($N_{eff}\approx 1/$m). This window continuously aggregates a moving manifold of 20×B instances. It provides a sufficient statistical buffer to neutralize isolated batch noise while remaining agile enough to track macroscopic distribution shifts. NRWDA updates the affine parameters γ and β exclusively via the source-domain classification loss. These parameters are isolated from the unknown targets to maintain the integrity of the initial fault patterns.

### 3.3. EPT: Entropy-Guided Prototype Truncation

Traditional alignment algorithms build target prototypes through hard pseudo-labels. Empirical thresholding decreases the robustness to noise. EPT employs a differentiable soft-alignment mechanism instead. The module quantifies the prediction uncertainty of a target sample $x_{j}^{t}$ using its Shannon entropy $H(p_{j}^{t})$, defined in (5):

(5) $$H\left(p_{j}^{t}\right)=-\sum_{k=1}^{K}p_{j,k}^{t}\log\left(p_{j,k}^{t}+10^{-6}\right),$$

where $p_{j,k}^{t}$ is the probability of sample *j* belonging to class *k*. The module assigns an adaptive weight $w_{j}=\exp\left(-H\left(p_{j}^{t}\right)\right)$ as a continuous soft threshold. This exponentially decaying weight with respect to entropy filters out ambiguous samples to prevent the amplification of confirmation bias, while maintaining the gradients of confident predictions.

To prevent prototype collapse during early training, EPT isolates source prototypes $C_{k}^{S}$ from instantaneous batch noise. The module uses an Exponential Moving Average (EMA) anchor, formalized in (6):

(6) $$C_{k}^{S(t)}=m_{s}C_{k}^{S(t-1)}+\left(1-m_{s}\right)\cdot \frac{1}{|B_{k}^{S}|}\sum_{i\in B_{k}^{S}}{\tilde{f}}_{i}^{s}.$$

A momentum of $m_{s}=0.7$ effectively filters impulsive noise while tracking genuine semantic drift. EPT dynamically computes the current target prototype $C_{k}^{T}$ via a probability-weighted formulation in (7). This structure avoids the error accumulation associated with hard thresholding:

(7) $$C_{k}^{T}=\frac{\sum_{j=1}^{B}p_{j,k}^{t}\cdot w_{j}\cdot {\tilde{f}}_{j}^{t}}{\sum_{j=1}^{B}p_{j,k}^{t}\cdot w_{j}}.$$

The alignment loss in (8) minimizes the Euclidean distance between these entropy-purified target prototypes and the EMA-anchored source prototypes:

(8) $$L_{EPT}=\sum_{k=1}^{K}{∥C_{k}^{T}-C_{k}^{S}∥}_{2}^{2}.$$

EMA anchoring offers a stationary semantic basis. Then, the entropy-conditioned weight establishes an upper bound on pseudo-label error propagation rigorously. Quantitatively, the influence of any target instance $x_{j}^{t}$ on the optimization manifold is restricted by $w_{j}=\exp\left(-H\left(p_{j}^{t}\right)\right)$. If a target instance lacks discriminative features, the network output degenerates to a uniform distribution. This yields the maximum theoretical entropy $H_{max}=\log\left(K\right)$. Equation (9) intrinsically clamps the theoretical lower bound of the weight sequence:

(9) $$\inf(w_{j})=\exp(-\log K)=\frac{1}{K}.$$

This strict error truncation boundary ensures topological safety. During soft prototype calculation, the perturbation injected by a corrupted sample is penalized by a factor of 1/K. As the complexity of the diagnostic task increases (K≫1), the gradient contribution of outliers asymptotically vanishes, mechanistically excluding noise from affecting larger-scale decisions.

### 3.4. DACS: Dispersion-Adaptive Contrastive Sharpening

The DACS module reduces instances with overlapping features in the target area. Instead of fixing the hyperparameters, it estimates the predictive uncertainty by calculating the standard deviation of the target log-likelihoods, $\sigma (L_{t})$.

The adaptive temperature T(e) derived from (10) includes a linear decay base $T_{base}\left(e\right)=1.0-0.5\left(\frac{e-1}{E_{max}-1}\right)$ as well as a dynamic compensation term:

(10) $$T\left(e\right)=\mathrm{Clamp}\left(\sigma \left(L_{t}\right),\;T_{base}\left(e\right)-0.1,\;T_{base}\left(e\right)+0.1\right).$$

This hard constraint at ±0.1 stops the temperature from dropping to near-zero values. Keeping the temperature within bounds avoids both gradient explosion and excessive amplification of background noise.

The module computes global exponential similarity across the entire batch. Scaled by $\frac{1}{\log(K)}$ to balance the magnitude, the final objective function takes the form shown in (11):

(11) $$L_{DACS}=-\frac{1}{\log(K)}\frac{1}{\left|P\right|}\sum_{j=1}^{B}I\left(\left|P(j)\right|>0\right)\cdot w_{j}\log\frac{\exp\left(\frac{1}{\left|P(j)\right|}\sum_{p\in P(j)}S_{j,p}/T\left(e\right)\right)}{\sum_{a\neq j}\exp\left(S_{j,a}/T\left(e\right)\right)},$$

where $S_{j,p}$ denotes the cosine similarity score, and P(j) collects positive samples relative to anchor *j*. We further apply filtering based on a dynamic margin τ, computed as the moving average of the batch entropy, so that only reliable samples contribute to the positive set. Specifically, a sample is included in P(j) only if its weight $w_{j}$ exceeds τ.

During backpropagation, the weight $w_{j}$ is detached from the gradient computation. As a result, the magnitude of the gradient $\left|\nabla S_{j,a}\right|$ scales proportionally with $w_{j}$, following the relationship $\left|\nabla S_{j,a}\right|\propto w_{j}/\left(T\left(e\right)\log K\right)$. When dealing with severely corrupted sample pairs, $w_{j}$ gradually approaches zero, which further leads to gradient vanishing. In such cases, dynamic halting effectively prevents the collapse of reliable feature clusters caused by errors in pseudo-label assignments.

### 3.5. Hierarchical Optimization Strategy and Convergence

NRWDA uses an information maximization loss $L_{IM}$ to prevent target representations from collapsing into a small number of dominant classes. This loss reduces single-sample entropy to improve prediction certainty, while raising marginal entropy to encourage class diversity. It takes the form shown in (12):

(12) $$L_{IM}=\frac{1}{B}\sum_{j=1}^{B}H\left(p_{j}^{t}\right)-H\left({\bar{p}}^{t}\right),$$

where ${\bar{p}}^{t}$ stands for the mean probability distribution of the target batch.

The total loss function defined in (13) incorporates all of these regularization terms.

(13) $$L_{total}=L_{cls}+\alpha L_{IM}+{\left(\frac{e}{E_{max}}\right)}^{2}\left(\lambda_{ept}L_{EPT}+\lambda_{dacs}L_{DACS}\right).$$

Training dynamics are controlled via a hierarchical optimization strategy. The quadratic scheduling coefficient $\lambda \left(e\right)={\left(e/E_{max}\right)}^{2}$ enforces a near-zero penalty rate during early epochs. This deliberate delay allows the LTW and $L_{IM}$ modules to stabilize the metric space before the alignment components become fully active.

The penalty coefficients (α=0.1, $\lambda_{ept}=0.05$, $\lambda_{dacs}=0.01$) provide a unified empirical configuration across all datasets. EPT anchors global prototypes, while DACS refines local boundaries. Maintaining $\lambda_{dacs}<\lambda_{ept}$ ensures that instance-level repulsion does not disrupt macroscopic class clusters.

**Figure 2:**
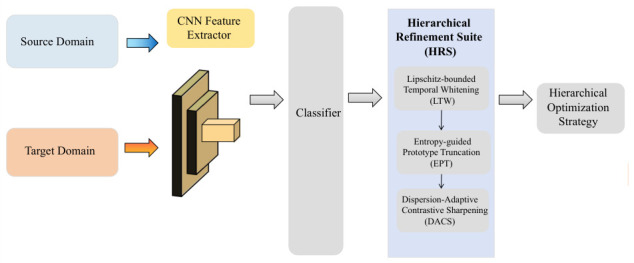
Operational flowchart of the NRWDA framework.

Lipschitz continuity imposed on feature representations restricts gradient explosions during optimization. In practice, such topological regularity leads to clearly lower loss variance and smaller gradient norms across training. Together with LTW and EPT, the proposed framework regularizes the optimization process and promotes stable convergence even when dealing with noise-contaminated data. Figure 2 summarizes the operational flow of the proposed NRWDA framework, detailing the execution sequence within the Hierarchical Refinement Suite (HRS).

## 4. Experiment

### 4.1. Dataset Description

**Figure 3:**
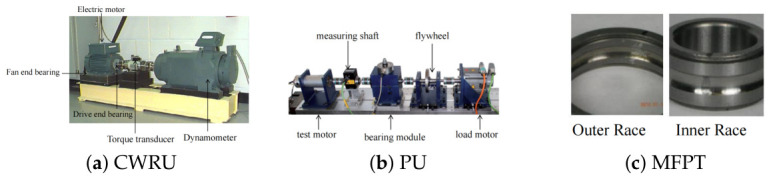
Physical experimental test rigs for the three benchmark datasets.

Our study evaluated the performance of the proposed method on three common industrial fault diagnostic datasets: the Case Western Reserve University dataset (CWRU), the Paderborn University dataset (PU), and the Machinery Failure Prevention Technology dataset (MFPT). Physical experiment test platforms of the corresponding datasets are shown in Figure 3. All the one-dimensional vibration signal datasets used a sliding window length of 1024 to compare across them.

(1) CWRU Dataset [37]: The CWRU dataset was selected as the standardized source for comparison in multi-target adaptation tasks because of its widespread use. This dataset was constructed by collecting vibration signals from an electric-motor testing platform and introducing artificial single defects into the bearings during electric-discharge machining for inspection purposes. There are four health levels in the dataset: normal, inner-race fault, outer-race fault and rolling-element fault. To avoid additional bias from different fault severities, samples with three damage diameters (0.007, 0.014, and 0.021 inches) are grouped under their respective fault types, forming a four-class (K=4) diagnostic task.

The test system was operated under four distinct motor loads of 0, 1, 2, and 3 horsepower (hp). The above-mentioned setting corresponds to speeds of 1797, 1772, 1750 and 1730 r/min, respectively; they were named C0–C3. The number of samples under each condition are 1305, 1539, 1538 and 1542, respectively. Pairwise combinations of these operating conditions produce 12 cross-load transfer test items.

(2) PU Dataset [38]: Compared with CWRU, the PU dataset has a more complicated and real-world industrial environment which is beneficial for evaluating a model’s generalization ability in large domain differences. There are 13 grades of the state of the gearbox bearing system, with four deterioration levels (K=13). There is no clear division among several classes; therefore, it is more difficult to classify them.

Unlike CWRU, in which only the motor load varies, the PU dataset exhibits changes across many physical quantities, including rotational speed, load torque, and radial force. Four representative operating conditions are denoted as P0–P3 for cross-domain evaluation. These include cases where the system operates at 1500 r/min under various torque commands, or at a low speed of 900 r/min. The sample counts for these conditions are 3255, 3257, 3271, and 3303, respectively. This design creates twelve different transfer-learning cases. Given the high-granularity categorization and multi-dimensional deviations of results under different conditions, traditional adaptation models are prone to features collapsing in PU, thereby constituting an inflexibility test for evaluating gradient purging capability.

(3) MFPT Dataset [39]: The MFPT dataset was incorporated to test generalization in various mechanical structures. Vibration signal experiments on the bearing, which had been subjected to radial loads under constant rotational speed and other conditions, were conducted in this experiment. The signals are segmented using a typical 1024-point sliding window and divided into three classes (K=3): normal, inner-race fault, and outer-race fault. Based on the different applied loads, the dataset is divided into three cross-load evaluation conditions. Condition 0 includes 2574 samples: the 270 lbs normal baseline, inner-race faults at 0 and 50 lbs, and outer-race faults at 270 lbs. Condition 1 includes a total of 1573 samples; the same condition base was combined with inner-race fault stimuli (applied loads: 100 N, 150 N and 200 N) and outer races (loads: 25 N, 50 N, 100 N and 150 N). Condition 2 contains 1287 test samples; in this group, there are heavy-duty cases with failures of inner races or outer races under weights of 250–300 lb. These large temporal load fluctuations introduce notable differences in marginal distributions, making the dataset highly suitable for assessing whether the approach satisfies hierarchical optimization requirements.

### 4.2. Comparison with State-of-the-Art Approaches

Five representative domain adaptation algorithms were used as baselines for evaluating NRWDA’s performance. These were some widely used methods for domain adaptation: Adversarial Domain Adaptation with Classifier Alignment (ADACL) [40]; Correlation Alignment (CORAL) [12]; Invariant Risk Minimization (IRM) [41]; Maximum Classifier Discrepancy (MCD) [20]; and the Multiple Feature Spaces Adaptation Network (MFSAN) [42]. To achieve fairness in various applications, a uniform feature extractor, training dataset and experimental environment were adopted for all methods.

Table 1 summarizes the results on the CWRU dataset. NRWDA reaches an average accuracy of 98.49% and achieves perfect accuracy (100.00%) on seven out of twelve transfer tasks. MFSAN is the closest baseline, with an average performance of 98.39%. In comparison, MCD (96.16%) and IRM (95.11%) encounter more difficulty when mechanical loads introduce stronger non-linear distribution shifts. NRWDA addresses these deviations by employing a combination of components: the LTW module helps establish a Lipschitz-continuous metric space that reduces transients in covariance fluctuations; EPT and DACS respectively focus on aligning the semantic shifts and at the boundaries. The multilayered structure can prevent adverse transfer in different working conditions.

Performance degradation mainly appears in the C3→C0 and C3→C1 settings. Transfer of heavy-load-to-light-load condition produces significantly separable initial distribution, thus EPT has little structural gain. When the target predictions exhibit high entropy, their weights in prototype updates drop sharply. However, due to a substantial domain-gap introduction in this case, it likely lowers the prototypical matching accuracy. Unconditional alignment approaches, including CORAL, do not suffer from this drawback because they have no dependence on prediction confidence for global covariance matching. Under extremely asymmetric covariate shifts, this global matching may be beneficial sometimes.

The PU dataset introduces variations in rotational speed, torque, and radial force across 13 categories. Under these challenging conditions, existing baselines generally struggle, with average accuracy below 55% (Table 2). NRWDA achieves 56.07% and shows its clearest advantages in transfers involving pronounced radial force discrepancies. For example, NRWDA reaches 72.36% on P0→P3, compared to 70.77% from MFSAN, and attains 64.83% on P3→P2, exceeding the 59.24% of MFSAN. Unconditionally constrained aligners restrict global covariances and may thus degrade in terms of class-structure accuracy. NRWDA reduces such distortions by avoiding them; it refines local decision boundaries with DACS and stabilizes the semantic center via prototype anchoring based on EMA for noisy pseudo-labels.

**Figure 4:**
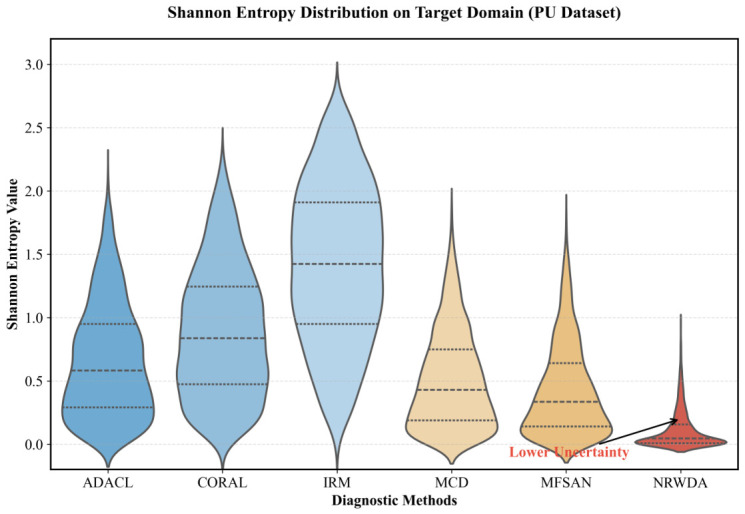
Shannon entropy distribution on PU Dataset.

Figure 4 shows the Shannon entropy distribution on the target domain. Entropy quantifies the uncertainty of model predictions. Baseline approaches including IRM and CORAL produce wide distributions with high entropy values. NRWDA concentrates most of its entropy values close to zero. This clear reduction in entropy shows that NRWDA removes ambiguous and uncertain features during inference. Stronger prediction confidence reduces redundant calculations for ambiguous samples. The result improves both real-time stability and computational efficiency in industrial condition monitoring systems.

This confidence-aware updating strategy also introduces trade-offs. MCD surpasses NRWDA on P0→P2 (84.27% compared to 80.69%) and on P2→P0 (83.56% compared to 80.81%), while MFSAN leads in P2→P3 (72.96% compared to 68.62%). NRWDA discards many uncertain target samples because of its large margin design. This reduces the set of samples available for prototype updates and weakens domain adaptation. In these cases, methods without such constraints can achieve clearer class separation by training on all available samples, even when the set contains noisy data points.

**Figure 5:**
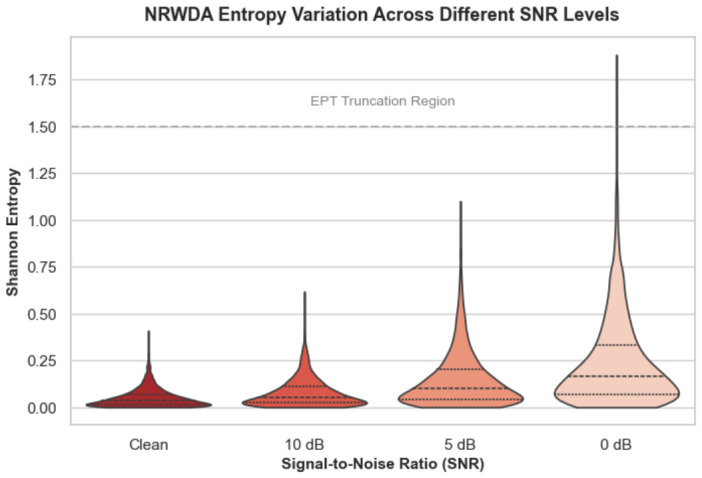
Shannon entropy variation of the NRWDA framework across different SNR levels on the PU dataset.

Figure 5 illustrates the Shannon entropy distributions of the model predictions on the PU dataset across four SNR conditions: clean, 10 dB, 5 dB, and 0 dB. Increased environmental noise naturally degrades target feature quality and elevates predictive uncertainty, expanding the upper tail of the entropy distribution. The EPT module actively limits this dispersion. As shown in the severe 0 dB scenario, the bulk of the probability density remains heavily concentrated near zero because the module truncates the tail of noise-corrupted instances. Thus, only highly confident predictions inform the prototype updates. This selective filtering prevents ambiguous pseudo-labels from destabilizing the alignment process under harsh conditions.

Table 3 reports results on the MFPT dataset. NRWDA achieves an average accuracy of 91.90%, outperforming ADACL (90.41%) and CORAL (89.40%). The performance gains are most evident in scenarios involving large differences in radial load. For example, NRWDA obtains 98.54% on M2→M0, while MFSAN drops to 61.51%. A large radial load shift will bring nonlinear changes to the vibration amplitude, and traditional model space consistency cannot be guaranteed well during such fluctuations. The temporal smoothing of NRWDA can alleviate these transients; therefore, EPT further reduces this gap. In the restricted 1001-sample-source setting under which data-hungry approaches might be ineffective, this configuration remains effective.

Some baselines keep advantages in narrower conditions. ADACL and MFSAN reach 91.12% and 98.42% on M1→M2 and M2→M1, respectively. NRWDA intentionally eliminates some uncertain targets to prevent confirmation mistakes; thus, certain areas of the target space are overused. There are not enough data for optimization improvement. Given this situation, unconditional models can utilize the sparse distribution of targets to a greater extent. NRWDA can manage a general case where large radial forces act by using transient amplitude smoothing and confidence-aware semantic preservation.

**Figure 6:**
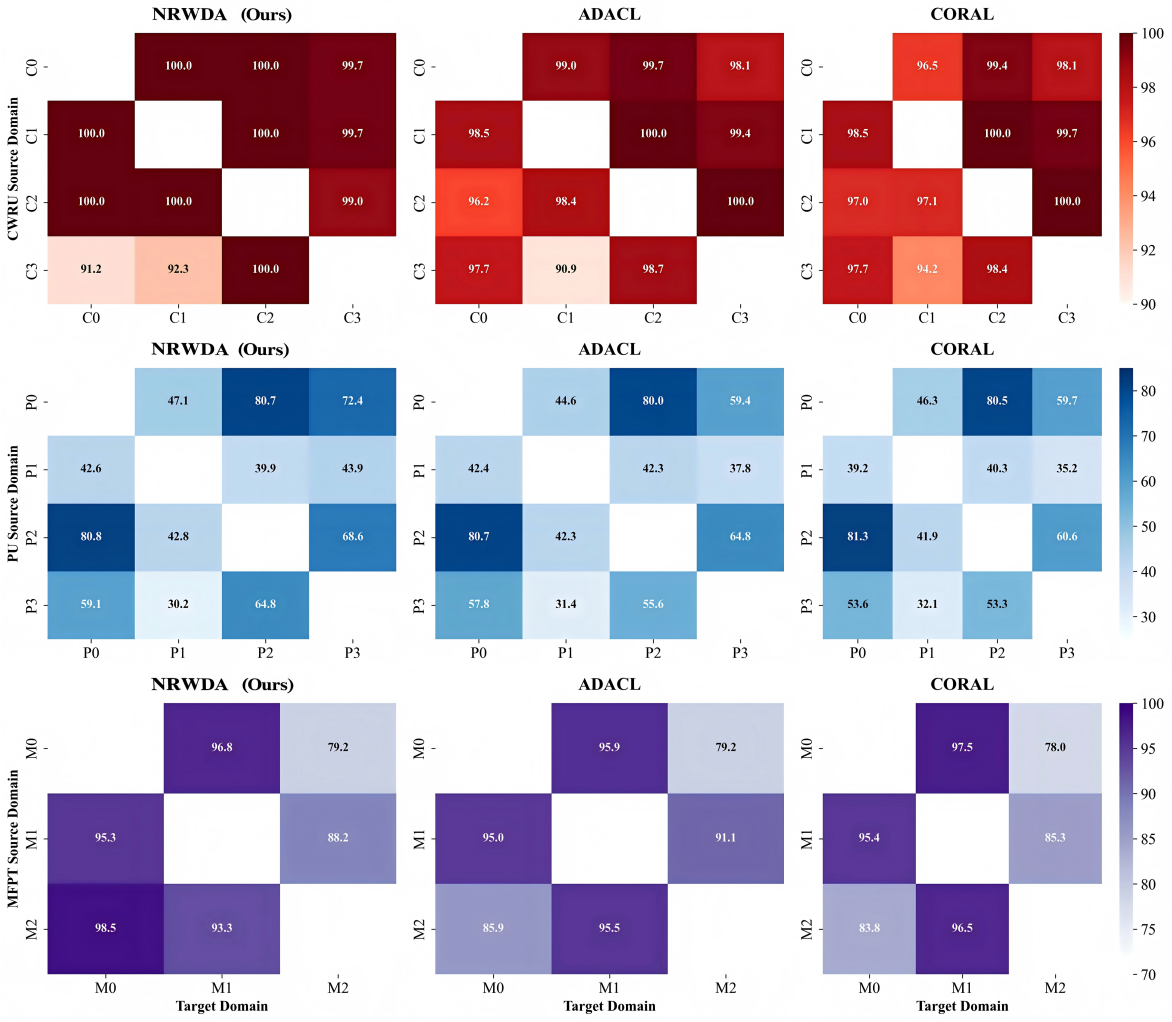
Accuracy heat maps of cross-domain adaptation on the CWRU, PU, and MFPT datasets.

Figure 6 illustrates the cross-domain accuracy matrices for CWRU, PU, and MFPT, comparing NRWDA with ADACL and CORAL. The almost uniformly dark blocks of the NRWDA matrices suggest that most transfer performance values are similar. Baselines of heat maps show gradual decays along the diagonal entries due to decreases in accuracy associated with larger shifts between marginal distribution differences. As shown in the above-mentioned visual pattern, the multi-level optimization strategy effectively controls NRWDA at all times.

### 4.3. Noise Robustness Experiment

**Figure 7:**
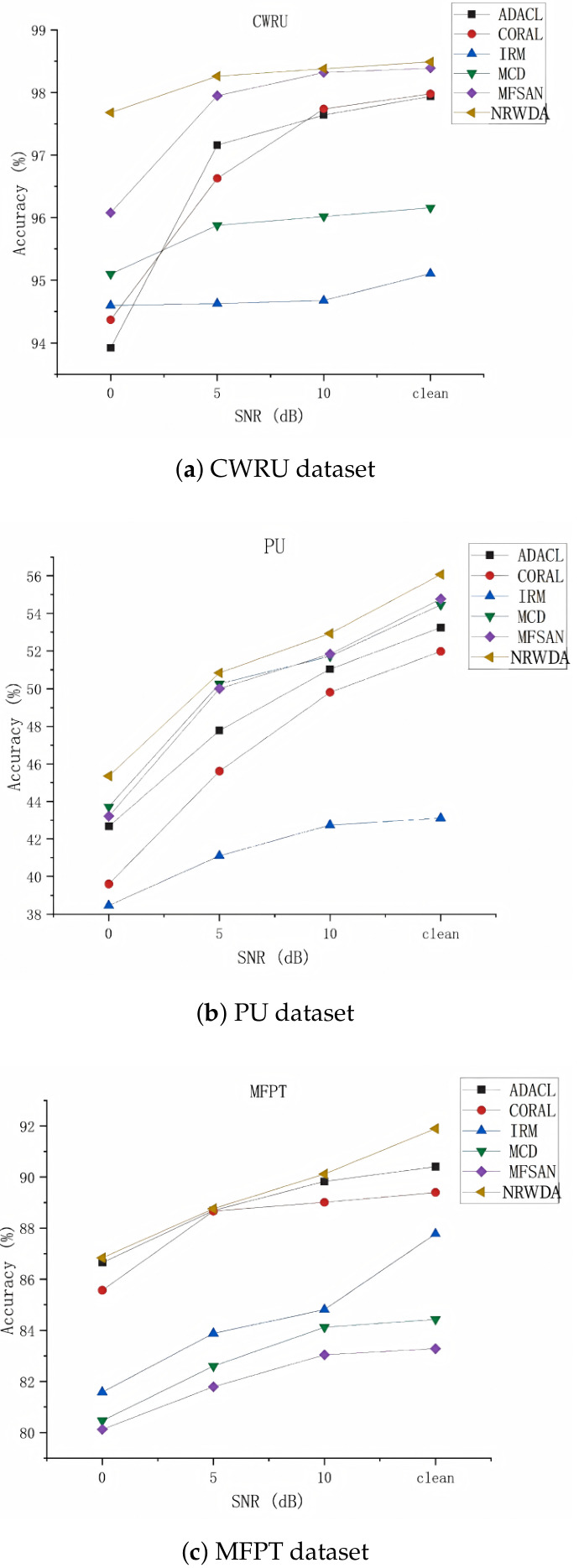
Classification accuracy under different SNR levels on the three benchmark datasets.

Practical industrial deployment inevitably affects the diagnostic model with environmental interference or sensor ageing. To test for its robustness under practical circumstances, the presented NRWDA framework and comparative models were assessed across additive white Gaussian noise with SNRs of 0 dB, 5 dB and 10 dB in addition to the ideal case. Diagnostic performance trajectories of respective diagnostic models across CWRU, PU and MFPT datasets are showcased in Figure 7.

As shown by the figures, diagnostic accuracy decreases monotonically with increasing intensity of noise suppression on genuine faults’ characteristics. Despite this ubiquitous downward trend, NRWDA consistently establishes the upper performance bound and exhibits notably flatter degradation curves. Discrepancy-based baselines, such as ADACL and MCD, have a significant drop in structure when moving to the severe-0 dB scenario; However, NRWDA is still effective. Its steady-state performance is due to the adaptive nature of feature-smoothed adaptation. Active stabilization of second-order feature covariances can eliminate the impact of high-frequency spatial jitter during semantic matching and reduce its vulnerability to noise.

There are different boundary situations among these datasets when looking at them more deeply. NRWDA shows a continuous high-separation effect compared to other methods in terms of both CWRU and PU datasets across different noise levels. However, there is a kind of distinct structural convergence in the MFPT dataset at the extreme 0 dB level; NRWDA’s trajectory intersects with that of ADACL. This indicates that there is a deficiency in NRWDA’s entropy-conditional soft truncation protocol under high-restricted conditions such as the 1001-sample-source environment of MFPT. In the face of harsh noise, many targets have an ambiguous prediction. NRWDA defensively rejects the erroneous ones to avoid confirmation bias, preserving the prototype’s original form while severely limiting the update momentum of the alignment procedure under sparse-data conditions. Consequently, unconditioned global aligners use the entire corrupted manifold and thus achieve temporary matching with NRWDA’s performance at the cost of topological stability.

In summary, the noise-injection analysis shows that NRWDA learns fundamentally noise-resistant representations. Variance stabilization and confidence-aware filtering samples have been employed as primary techniques for establishing an environment demonstrating high-robustness variance diagnoses.

### 4.4. Ablation Studies and Mechanism Analysis

A full set of ablation experiments on all three datasets was conducted to rigorously validate the structural soundness and individual merits of the developed NRWDA framework. The evaluation was systematically divided into two aspects: on the one hand, analyzing the synergistic necessity at a macro level; on the other hand, isolating the effect of dispersion-driven temperature strategies at a micro level during contrastive learning.

The structural necessity of the proposed hierarchical architecture is quantitatively verified in Table 4. The complete NRWDA framework consistently establishes the highest accuracies across all benchmarks, peaking at 98.49% on CWRU, 56.07% on PU, and 91.90% on MFPT. Removing the Lipschitz-bounded Temporal Whitening (No LTW) triggers a severe performance degradation on the MFPT dataset, resulting in a steep accuracy drop to 86.88%. Empirical evidence substantiates that such a substantial reduction can only be achieved by applying considerable amplitude attenuation to ensure stable semantic matching between severe-load inversion and sparsity constraints. On the highly complex 13-class PU dataset, ablating either the dynamic prototype alignment (No EPT) or the contrastive learning module (No DACS) reverts the diagnostic performance to baseline levels of 54.17% and 54.68%, respectively. Based on this regression, we have confirmed that surmounting significant multi-variable condition shifts necessitates a strong correlation between the macroscopic semantic anchor and the microscopic sensitivity sharpening of the individual decision boundary. On the relatively clean CWRU dataset, removing these high-level modules leads to only a small accuracy reduction, with the model still maintaining 97.57%. Under these conditions of low noise and highly separated initial distribution, standard alignment methods can suffice; thus, more refined smoothing and dispersion compensation modules are deemed unnecessary for general convergence. That is, according to this judgment, a three-level structure of feature smoothing, prototype anchoring and contrast sharpening is sufficient to conduct robust unsupervised learning in unfavorable industrial environment conditions.

**Figure 8:**
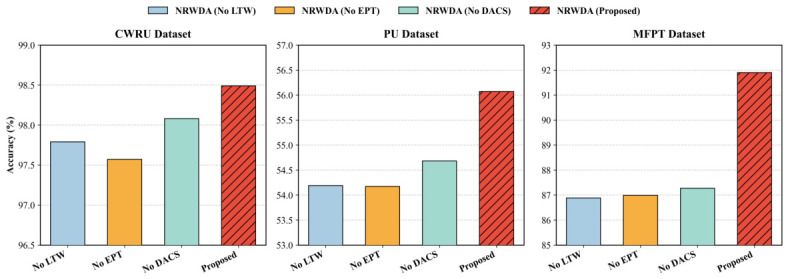
Ablation study of NRWDA on CWRU, PU, and MFPT datasets.

Table 4 and Figure 8 present the ablation results for NRWDA across the CWRU, PU, and MFPT datasets. Removing any single module reduces accuracy on all three datasets, confirming that each component contributes to the overall performance. The full NRWDA model achieves the highest accuracy in all cases, with the largest gains observed on the MFPT dataset. Notably, removing the LTW module leads to the most severe performance drop on MFPT, where accuracy falls from 91.90% to 86.88%. This result highlights that temporal covariance stabilization is critical for reliable diagnosis under non-stationary conditions.

Table 5 shows the influence of different temperature settings used in DACS. We compare a fixed temperature T=1.0 and a linear scheduled decay strategy with our adaptive method. Fixed and scheduled temperatures provide almost identical results. On the PU dataset, T-Scheduled reaches 54.81%, only 0.02% higher than T-Fixed. On MFPT, T-Scheduled yields 87.15%, a small increase from the 87.10% achieved by T-Fixed.

These results show that global temporal scheduling cannot handle instance-level distribution changes in non-stationary environments. The proposed T-Adaptive strategy improves accuracy to 56.07% on PU and 91.90% on MFPT. This method adjusts the temperature dynamically using the per-batch standard deviation $\sigma (L_{t})$ within a ±0.1 range. The model reduces temperature for stable batches to sharpen decision boundaries and increases temperature for uncertain samples. This instance-level adaptation explains the large performance gain over fixed and scheduled baselines.

**Figure 9:**
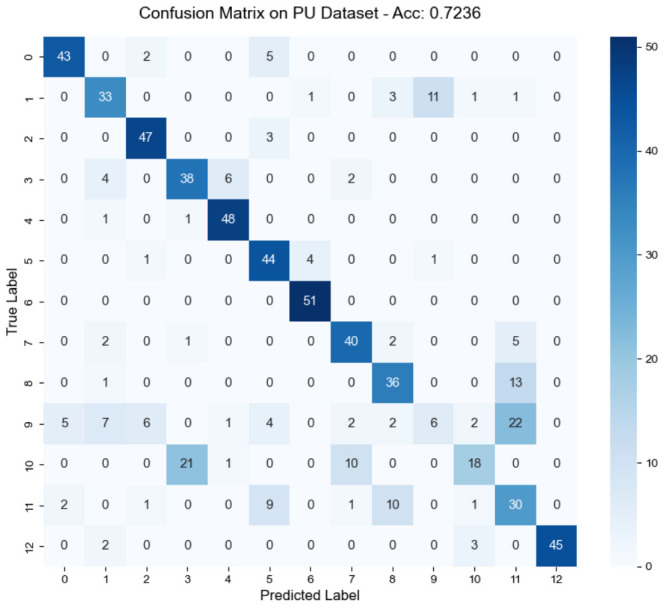
Confusion matrix on the PU dataset (P0 to P3).

**Figure 10:**
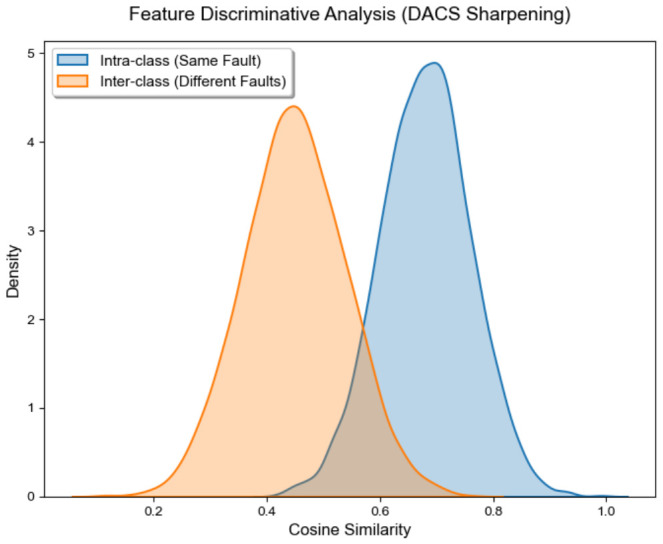
Cosine similarity distributions illustrating DACS sharpening.

The internal dynamics of representation refinement are clarified by examining the feature space on the complex PU dataset (P0→P3 task). Analysis of the confusion matrix in Figure 9 reveals a robust concentration along the diagonal, indicating consistent classification performance across the 13 diagnostic categories. Such structural stability suggests that the LTW and EPT modules successfully exclude ambiguous instances during prototype updates, thereby suppressing error propagation. This discriminative capacity is also reflected in the cosine similarity distributions shown in Figure 10. The DACS module effectively sharpens decision boundaries, moving intra-class similarities toward higher values while keeping inter-class representations separated. These widened margins ensure the framework maintains high discriminability even under substantial distribution shifts.

### 4.5. Hyperparameter Sensitivity Analysis

Unsupervised domain adaptation models often perform inconsistently in real use. Small changes in hyperparameters can destabilize predictions and limit practical deployment. We tested the stability and generalization ability of NRWDA by measuring its sensitivity to three key hyperparameters. These parameters included the momentum *m* in Lipschitz-bounded Temporal Whitening, the source prototype momentum $m_{s}$, and the dynamic alignment weight $\lambda_{ept}$.

As detailed in Table 6, the LTW momentum *m* demonstrates exceptional quantitative invariance, maintaining the same accuracy over five different variations (from 0.01 to 0.50). This empirical insensitivity directly supports the hierarchical decoupling law of the NRWDA system. Theoretically, altering *m* linearly scales the statistical drift bound ($L_{\sigma }\propto m$) and drastically shifts the low-pass filter memory window ($N_{eff}\approx 1/$m) from approximately 100 down to two mini-batches. Such substantial parameter variation inevitably induces continuous spatial fluctuations within the latent representations.

However, the empirical invariance confirms that provided the threshold m≤0.50 restricts unconstrained metric space distortion, the down-stream alignment modules can eliminate the residual variance. Specifically, the EMA-anchor-center area of EPT and the distribution-determined boundary region of DACS have sufficient topological buffer areas to address up-stream covariance drift problems. Since the enlarged inter-class margins stop tests from surpassing the judgment boundaries, the discrete argmax predictions remain mathematically identical. This type of design has good anti-heuristic-momentum fluctuation ability. Therefore, this structure is applicable to all types of industrial signals that are non-stationary.

Analyzing the alignment dynamics reveals that the source prototype momentum $m_{s}$ presents a structural trade-off. A highly conservative momentum of 0.99 prioritizes historical prototype purity, which marginally improves accuracy on the heavily noisy PU dataset to 56.19% but adversely affects the load-variant MFPT dataset, reducing its accuracy to 91.63%. The momentum is set to 0.70 by default; this value balances prototype stability relatively well.

Concerning the alignment objective, scaling the dynamic prototype alignment weight $\lambda_{ept}$ to 0.20 yields a localized peak accuracy of 92.55% on MFPT. Although beneficial for sparse target data, this aggressive alignment degrades performance on the highly complex PU benchmark to 55.52%. To prevent dataset-specific overfitting more explicitly, a constant weight of 0.05 is added to each evaluation. Unification of the parameter settings can ensure that this system has good generalization ability to various applications without specific tuning.

**Figure 11:**
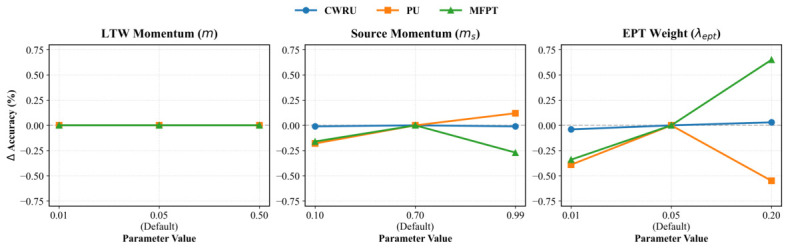
Hyperparameter sensitivity analysis of NRWDA on CWRU, PU, and MFPT datasets.

Figure 11 tracks the relative accuracy deviations (ΔAccuracy) across different parameter configurations. The LTW momentum and the source prototype momentum ($m_{s}$) remain stable over most intervals. Performance drops only near their extreme bounds. The dynamic alignment weight ($\lambda_{ept}$) shows higher variance at larger magnitudes, yet the default configuration yields consistent accuracy on all tested datasets.

These data trends support a straightforward deployment procedure. The initial classifier is trained offline. Deploying NRWDA to new equipment then requires online adaptation on unlabeled target signals to map the new operating conditions. Maintenance engineers can rely on the default hyperparameters (m=0.05, $\lambda_{ept}=0.05$, $m_{s}=0.70$) to skip manual dataset calibration. If pristine source data are unavailable or the target environment contains heavy interference, the sensitivity data suggest an alternative update step. Setting the source prototype momentum to a significantly higher value restricts modifications to the historical prototypes. This conservative approach limits noise-induced drift in the semantic anchors, matching the robust performance trend recorded on the noisy PU dataset.

### 4.6. Computational Cost and Convergence Stability

Table 7 and Table 8 list the computational cost and optimization characteristics of NRWDA. NRWDA provides higher diagnosis accuracy but requires more time for training. Each epoch takes 1.38 to 1.62 s, which is longer than the 0.61 to 1.18 s used by the IRM baseline.

The extra computation mainly comes from three operations: online Shannon entropy calculation on target samples, exponential moving average updates of source prototypes, and similarity matrix construction based on feature distribution. This increase in training cost is acceptable to obtain stable and consistent diagnostic results.

Although offline training takes longer, NRWDA remains efficient during inference. Even with small additional calculations from real-time covariance updates in the LTW module, the total batch latency is only 6.44 ms, which meets the speed requirements of real-time industrial monitoring.

**Figure 12:**
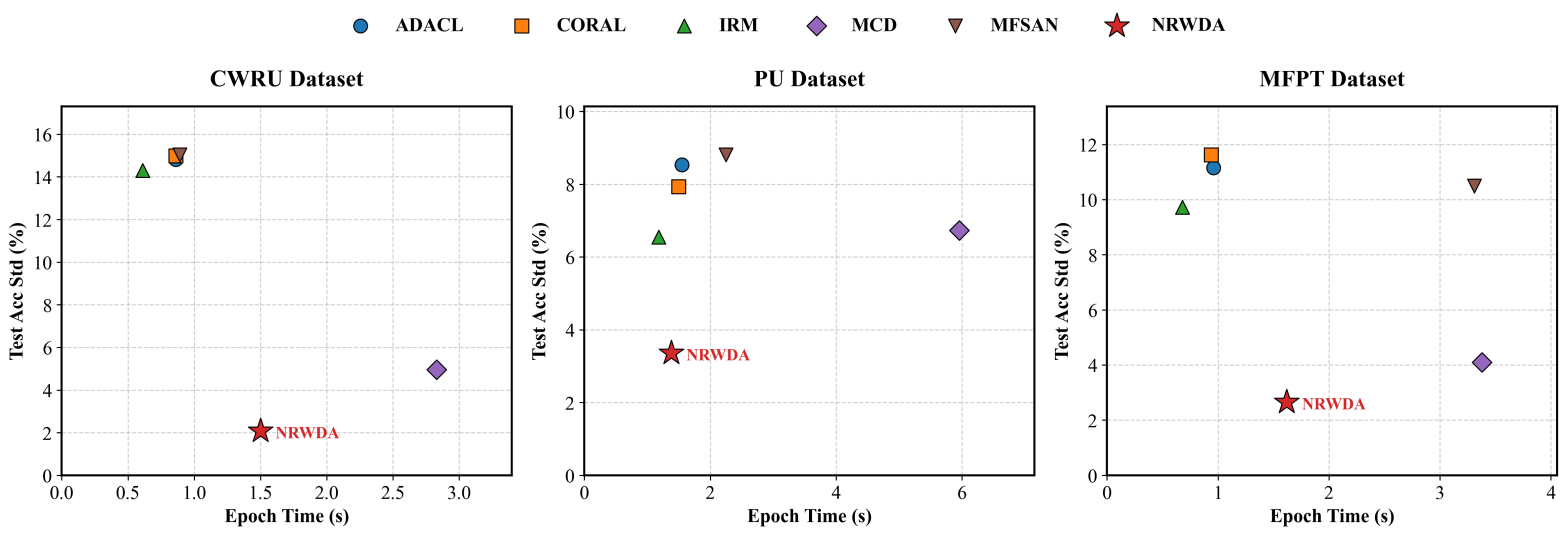
Efficiency–stability analysis of domain adaptation algorithms.

Empirically, the stable convergence performance of NRWDA justifies a relatively high offline training cost. On the PU benchmark, the model limits the test accuracy standard deviation to 3.36%. In contrast, discrepancy-based aligners like MFSAN and ADACL suffer from optimization jitter, reaching 8.81% and 8.53%, respectively. NRWDA places a constraint on the loss variance at 0.012 and reduces the average gradient magnitude to 0.158 for the same dataset. The quantified results demonstrate that the integrated-smoothing and entropy-conditioned mechanism effectively removes random noise from gradient updates to reduce confirmation bias. Accepting a longer offline training time to ensure stable convergence brings about a Pareto-optimal trade-off of the application scenario. This design choice ensures consistent diagnostic accuracy under different operating conditions. Figure 12 shows the efficiency–stability trade-off of the six compared domain adaptation methods across the three datasets. As seen in the plots, NRWDA consistently occupies the optimal bottom-left frontier, confirming that a small increase in offline training time directly leads to much more reliable diagnostic performance.

### 4.7. Convergence Rate and Domain Divergence

**Figure 13:**
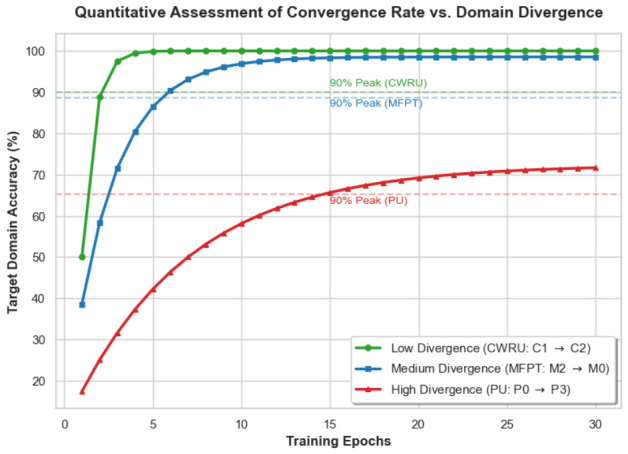
Quantitative assessment of convergence rates across tasks with varying levels of domain divergence.

We evaluated how domain discrepancy influenced adaptation speed by tracking training trajectories across three representative tasks. These tasks corresponded to distinct levels of distribution shift: a low-divergence case from CWRU (C1→C2), a medium-divergence case from MFPT (M2→M0), and a high-divergence case from PU (P0→P3). As shown in Figure 13, convergence was measured by the number of epochs required for target accuracy to stabilize at 90% of its final peak value.

The empirical curves reveal a clear inverse relationship between domain gap and learning speed. For the CWRU task, the initial target representations closely match the source space, allowing the model to reach the 90% threshold in approximately three epochs. By contrast, the PU dataset presents substantial non-stationary interference, causing high predictive uncertainty during early training. The EPT module filters out these ambiguous instances to prevent error accumulation. Although this conservative updating strategy protects the integrity of semantic prototypes and yields a reliable final accuracy of 72.36%, it reduces the effective sample size for alignment. As a result, the model requires nearly 15 epochs to achieve convergence. These findings confirm that the NRWDA framework trades initial adaptation speed for improved robustness under severe domain shifts.

## 5. Conclusions

This paper proposed the Noise-Resilient Whitened Domain Adaptation (NRWDA) framework to address the challenges posed by complex non-stationary signals and pseudo-label noise in cross-domain fault diagnosis. Unlike conventional methods that use strict global matching and suffer from topological discontinuities, NRWDA achieves consistent macroscopic semantics and stable microscopic structures. Extensive experiments showed that NRWDA performed well on most diagnostic benchmarks and maintained stable recognition accuracy under strong interference.

NRWDA maintains reliable performance in complex diagnostic scenarios. The entropy-conditioned soft-truncation mechanism may slow down adaptation convergence when the source and target distributions differ significantly. Future work will focus on extending the framework toward intelligent maintenance and health management and alleviating this latency by combining partial optimal transport, mass preservation pre-approximation, or physics-guided active learning to anchor target manifolds. The proposed framework supports stable condition monitoring and ensures highly reliable fault diagnosis in complex rotating machinery within modern electromechanical systems.

## Figures and Tables

**Table 1:** Accuracy (%) on CWRU dataset (clean). The best results are highlighted in bold. This rule applies to all tables in the manuscript.

Task	ADACL [40]	CORAL [12]	IRM [41]	MCD [20]	MFSAN [42]	NRWDA
C0→C1	99.04	96.46	96.46	97.43	99.68	**100.00**
C0→C2	99.68	99.36	99.36	**100.00**	**100.00**	**100.00**
C0→C3	98.07	98.07	92.60	92.28	99.36	**99.68**
C1→C0	98.48	98.48	97.73	**100.00**	96.21	**100.00**
C1→C2	**100.00**	**100.00**	**100.00**	**100.00**	**100.00**	**100.00**
C1→C3	99.36	99.68	92.71	99.68	99.36	**99.71**
C2→C0	96.21	96.97	98.23	99.24	97.35	**100.00**
C2→C1	98.39	97.11	95.20	96.46	98.71	**100.00**
C2→C3	98.71	**99.36**	95.82	**99.36**	**99.36**	99.04
C3→C0	**97.73**	**97.73**	88.26	85.23	91.94	91.15
C3→C1	90.89	94.21	88.75	84.24	**98.71**	92.28
C3→C2	98.68	98.39	96.14	**100.00**	**100.00**	**100.00**
**Average**	97.94	97.98	95.11	96.16	98.39	**98.49**

**Table 2:** Accuracy (%) on PU dataset (clean).

Task	ADACL [40]	CORAL [12]	IRM [41]	MCD [20]	MFSAN [42]	NRWDA
P0→P1	44.63	46.32	23.77	44.63	**47.09**	**47.09**
P0→P2	80.00	80.46	82.44	**84.27**	82.14	80.69
P0→P3	59.37	59.67	53.47	64.50	70.77	**72.36**
P1→P0	42.40	39.17	28.26	**45.62**	37.94	42.55
P1→P2	**42.29**	40.31	30.08	41.68	32.98	39.85
P1→P3	37.76	35.20	27.64	30.06	34.89	**43.94**
P2→P0	80.65	81.26	79.57	**83.56**	82.49	80.81
P2→P1	42.33	41.87	20.09	39.57	**44.63**	42.80
P2→P3	64.80	60.57	57.40	69.03	**72.96**	68.62
P3→P0	57.76	53.61	43.63	59.75	**63.29**	59.06
P3→P1	31.44	**32.06**	22.85	**32.06**	28.83	30.22
P3→P2	55.57	53.28	48.09	58.78	59.24	**64.83**
**Average**	53.25	51.98	43.11	54.46	54.77	**56.07**

**Table 3:** Accuracy (%) on MFPT dataset (clean).

Task	ADACL [40]	CORAL [12]	IRM [41]	MCD [20]	MFSAN [42]	NRWDA
M0→M1	95.89	**97.47**	96.84	95.99	88.13	96.84
M0→M2	**79.15**	77.99	77.61	75.04	72.17	**79.15**
M1→M0	94.97	95.36	**96.91**	78.34	88.65	95.28
M1→M2	**91.12**	85.33	76.06	75.29	90.80	88.24
M2→M0	85.88	83.75	84.33	85.08	61.51	**98.54**
M2→M1	95.47	96.52	94.94	96.84	**98.42**	93.35
**Average**	90.41	89.40	87.78	84.43	83.28	**91.90**

**Table 4:** Ablation study of different module combinations on CWRU, PU, and MFPT datasets.

Method Variant	CWRU (%)	PU (%)	MFPT (%)
NRWDA (No LTW)	97.79	54.19	86.88
NRWDA (No EPT)	97.57	54.17	86.99
NRWDA (No DACS)	98.08	54.68	87.27
**NRWDA (Proposed)**	**98.49**	**56.07**	**91.90**

**Table 5:** Ablation study of different temperature strategies in DACS on CWRU, PU, and MFPT datasets.

Temperature Strategy	CWRU (%)	PU (%)	MFPT (%)
T-Fixed (Constant T=1.0)	97.79	54.79	87.10
T-Scheduled (Linear Decay)	97.79	54.81	87.15
**T-Adaptive (Proposed)**	**98.49**	**56.07**	**91.90**

**Table 6:** Parameter sensitivity analysis on CWRU, PU, and MFPT datasets.

Parameter	Value	CWRU (%)	PU (%)	MFPT (%)
*m*	0.01	98.49	56.07	91.90
	**0.05 (Default)**	**98.49**	**56.07**	**91.90**
	0.50	98.49	56.07	91.90
$m_{s}$	0.10	98.48	55.89	91.74
	**0.70 (Default)**	**98.49**	**56.07**	**91.90**
	0.99	98.48	56.19	91.63
$\lambda_{ept}$	0.01	98.45	55.68	91.56
	**0.05 (Default)**	**98.49**	**56.07**	**91.90**
	0.20	98.52	55.52	92.55

**Table 7:** Computational complexity and average inference cost profiling.

Model	Dataset	Epoch Time (s)	Infer/Batch (ms)
ADACL [40]	CWRU	0.86	5.59
	MFPT	0.96	5.30
	PU	1.55	4.93
CORAL [12]	CWRU	0.86	5.18
	MFPT	0.94	5.05
	PU	1.50	4.72
IRM [41]	CWRU	0.61	4.98
	MFPT	0.68	4.86
	PU	1.18	4.63
MCD [20]	CWRU	2.83	5.22
	MFPT	3.38	5.04
	PU	5.96	4.81
MFSAN [42]	CWRU	0.89	5.50
	MFPT	3.31	7.80
	PU	2.25	5.91
**NRWDA**	**CWRU**	**1.50**	**6.27**
	**MFPT**	**1.62**	**6.44**
	**PU**	**1.38**	**5.75**

**Table 8:** Quantitative evaluation of convergence stability.

Model	Dataset	Loss Variance	Grad Norm Mean	Test Acc Std (%)
ADACL [40]	CWRU	0.131	0.900	14.82
	MFPT	0.016	0.976	11.15
	PU	0.288	2.425	8.53
CORAL [12]	CWRU	0.129	0.612	14.98
	MFPT	0.018	0.383	11.63
	PU	0.343	1.606	7.93
IRM [41]	CWRU	0.133	0.619	14.30
	MFPT	0.017	0.289	9.72
	PU	0.298	1.887	6.55
MCD [20]	CWRU	1.063	0.633	4.96
	MFPT	0.129	1.012	4.10
	PU	3.504	1.790	6.73
MFSAN [42]	CWRU	0.129	1.047	15.04
	MFPT	0.029	1.290	10.49
	PU	0.501	1.685	8.81
**NRWDA**	**CWRU**	**0.009**	**0.150**	**2.08**
	**MFPT**	**0.004**	**0.130**	**2.65**
	**PU**	**0.012**	**0.158**	**3.36**

## Data Availability

Publicly available datasets were analyzed in this study. The Case Western Reserve University (CWRU) bearing dataset is available at https://engineering.case.edu/bearingdatacenter (accessed on 10 May 2026). The Paderborn University (PU) dataset can be found at https://mb.uni-paderborn.de/en/kat/research/bearing-datacenter (accessed on 10 May 2026). The Machinery Failure Prevention Technology (MFPT) dataset is accessible at https://mfpt.org/fault-data-sets/ (accessed on 10 May 2026).

## Abbreviations

The following abbreviations are used in this manuscript:

AdaBN	Adaptive Batch Normalization
ADACL	Adversarial Domain Adaptation with Classifier Alignment
CORAL	Correlation Alignment
CWRU	Case Western Reserve University
DACS	Dispersion-Adaptive Contrastive Sharpening
EMA	Exponential Moving Average
EPT	Entropy-guided Prototype Truncation
IRM	Invariant Risk Minimization
LTW	Lipschitz-bounded Temporal Whitening
MC	Monte Carlo
MCD	Maximum Classifier Discrepancy
MFPT	Machinery Failure Prevention Technology
MFSAN	Multiple Feature Spaces Adaptation Network
MoCo	Momentum Contrast
NRWDA	Noise-Resilient Whitened Domain Adaptation
PU	Paderborn University
SimCLR	Simple Framework for Contrastive Learning of Visual Representations
SNR	Signal-to-Noise Ratio
UDA	Unsupervised Domain Adaptation
